# Chlorin e6 – polyvinylpyrrolidone mediated photosensitization is effective against human non-small cell lung carcinoma compared to small cell lung carcinoma xenografts

**DOI:** 10.1186/1471-2210-7-15

**Published:** 2007-12-01

**Authors:** William WL Chin, Paul WS Heng, Malini Olivo

**Affiliations:** 1 Division of Medical Sciences, 11 Hospital Drive, Singapore 169610, National Cancer Centre, Singapore; 2 Department of Pharmacy, National University of Singapore; No. 18 Science Drive 4, Block S4, Singapore 117543, Singapore

## Abstract

**Background:**

Photodynamic therapy (PDT) is an effective local cancer treatment that involves light activation of a photosensitizer, resulting in oxygen-dependent, free radical-mediated cell death. Little is known about the comparative efficacy of PDT in treating non-small cell lung carcinoma (NSCLC) and small cell lung carcinoma (SCLC), despite ongoing clinical trials treating lung cancers. The present study evaluated the potential use of chlorin e6 – polyvinylpyrrolidone (Ce6-PVP) as a multimodality photosensitizer for fluorescence detection and photodynamic therapy (PDT) on NSCLC and SCLC xenografts.

**Results:**

Human NSCLC (NCI-H460) and SCLC (NCI-H526) tumor cell lines were used to establish tumor xenografts in the chick chorioallantoic membrane (CAM) model as well as in the Balb/c nude mice. In the CAM model, Ce6-PVP was applied topically (1.0 mg/kg) and fluorescence intensity was charted at various time points. Tumor-bearing mice were given intravenous administration of Ce6-PVP (2.0 mg/kg) and laser irradiation at 665 nm (fluence of 150 J/cm^2 ^and fluence rate of 125 mW/cm^2^). Tumor response was evaluated at 48 h post PDT. Studies of temporal fluorescence pharmacokinetics in CAM tumor xenografts showed that Ce6-PVP has a selective localization and a good accuracy in demarcating NSCLC compared to SCLC from normal surrounding CAM after 3 h post drug administration. Irradiation at 3 h drug-light interval showed greater tumor necrosis against human NSCLC xenografts in nude mice. SCLC xenografts were observed to express resistance to photosensitization with Ce6-PVP.

**Conclusion:**

The formulation of Ce6-PVP is distinctly advantageous as a diagnostic and therapeutic agent for fluorescence diagnosis and PDT of NSCLC.

## Background

Photodynamic therapy (PDT) is a promising modality in both the curative and palliative treatment against a variety of experimental and naturally occurring human cancers [1]. Essentially, PDT is a two-step process that begins with the administration of photosensitizer for selective uptake in the target tissue. The second phase involves exposure to non-thermal light at a wavelength specific to the photosensitizer at the sensitized target tissue. The activation of the photosensitizer by light is an oxygen-dependent process that results in the generation of highly cytotoxic species including singlet oxygen. The release of these reactive molecules results in damage to both tumor cells and to the tumor microenvironment. The significance of PDT is that there is a degree of treatment selectivity that allows tumor destruction with minimal involvement of healthy tissue. This is achieved by a combination of selective accumulation of photosensitizer within the tumor and by control of the light geometry and illumination parameters [2].

Lung cancer became one of the first cancers to be considered for PDT and has been used as an adjuvant treatment over the last 27 years [3]. Currently, PDT is used either to treat microinvasive endobronchial non-small cell lung carcinoma (NSCLC) or to palliate patients with completely or partially obstructing endobronchial NSCLC [4]. Despite the generally refractory nature of these type of tumors, central type of tumors with identifiable endobronchial lesions which could be easily accessed bronchoscopically for illumination have been successfully treated with PDT [5]. PDT can preserve lung function, limiting surgical trauma and postoperative pain as well as used in combination with other therapeutic modalities such as chemotherapy [4]. Photosensitizer-induced fluorescence detection aimed at enhancing optical contrast to improve tumor visibility has been extensively investigated to develop 'tumor selective' imaging methods [6,7]. The lack of tumor selectivity, complex pharmacokinetics and the fact that some photosensitizers may cause prolonged skin photosensitivity, make the clinical application of fluorescence detection and PDT more complex [8,9]. These limitations have led to the development of second-generation photosensitizers, which usually produce shorter periods of photosensitivity, longer activation wavelengths, higher tumor-to-normal tissue concentration, excellent antitumor effect and higher quantum yields of ^1^O_2 _[10]. Studies showed that derivatives from chlorophylls/chlorins are potent photosensitizers [11,12], of which mono-L-aspartyl chlorin e6 (NPe6, Laserphyrin) is undergoing clinical trials in Japan for the treatment of endobronchial lung cancer [13].

**Figure 1:**
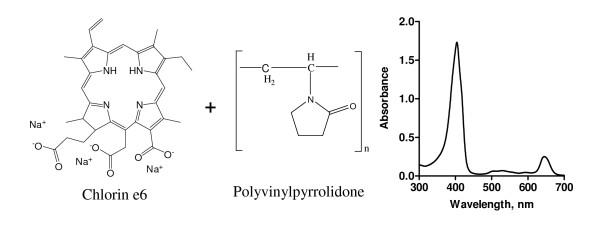
**Molecular structure of Ce6, PVP and the absorption spectra of Ce6-PVP in PBS measured from 400 to 800 nm**. Ce6-PVP has a prominent absorption at 400 nm and 665 nm.

This report investigates a new formulation that consists of a mixture of chlorin e6 (Ce6) derived from the plant *Spirullina platensis *and polyvinylpyrrolidone (PVP, molecular mass = 12,000) (Fig 1). PVP is a biocompatible hydrophilic polymer that has been used to improve dissolution of lipophilic drugs and to modify the biodistribution of the drug. The mixture of Ce6 and PVP has a mass fraction ratio of 1:1. Ce6-PVP absorbs light of wavelength above 665 nm and produces less long-term normal tissue phototoxicity than Photofrin [14]. In our previous studies, we have demonstrated that Ce6-PVP selectively accumulated in the poorly differentiated human nasopharyngeal and human bladder carcinoma xenografts in animal models [15-17]. We have also reported the potential application of Ce6-PVP in photodynamic therapy in one angiosarcoma patient [18]. The present work examines the fluorescence pharmacokinetic of Ce6-PVP in NSCLC and small cell lung carcinoma (SCLC) xenografts on the chick chorioallantoic membrane (CAM) model. We have applied the receiver operating characteristics (ROC) concept to compare the sensitivity and specificity of fluorescence imaging on NSCLC and SCLC using white light as the gold standard. Photosensitizing efficacy of Ce6-PVP was also investigated between the two histology of lung carcinoma using murine xenografts model.

## Results and discussion

**Figure 2:**
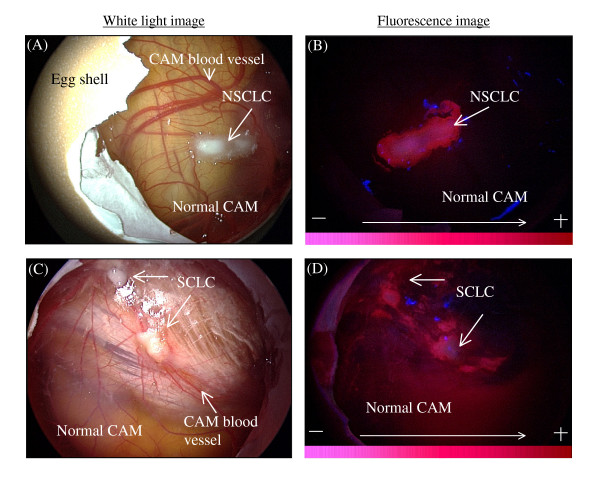
**Fluorescence imaging of lung cancers xenografted on the CAM model**. Representative of white light images of NSCLC and SCLC grafted on CAM before administration of photosensitizer (Fig 2A, B). Before incubation of Ce6-PVP, the CAM tumor xenografts were imaged under blue light illumination, to confirm that there was no autofluorescence. Tumor fluorescence images at 3 h post-topical administration of 1 mg/kg of Ce6-PVP under blue light illumination (Fig 2C, D).

**Figure 3:**
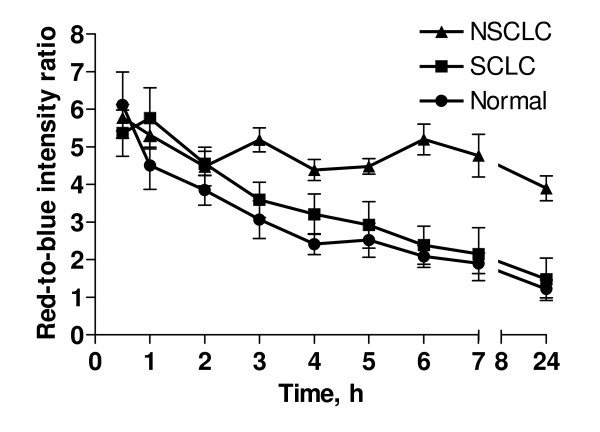
**Fluorescence kinetics of Ce6-PVP on NSCLC (▲) and SCLC (■) xenografted on CAM examined up to 24 h post topical drug administration**. Values are expressed as red-to-blue intensity ratio of fluorescence images post administration of drug normalized with images before drug administration. For tumor, each point represents a mean of 5 eggs whereas for normal (●), each point represents a mean of 10 eggs. Bars = standard error of the mean. Non-linear regression analysis demonstrated that all the curves were statistically different with each other. The elimination rate constant for NSCLC, SCLC and normal CAM was in the following order: NSCLC < SCLC < normal CAM.

Fluorescence bronchoscopy has been reported to enhance the diagnostic accuracy and definition of the intra-epithelial cancer within the bronchi [19]. This technique has become more attractive for clinical use since more effective 2nd generations of photosensitizers have been clinically implemented and tested. Newer formulations of photosensitizers were intended to reduce common side effects such as skin photosensitivity, nausea, vomiting and transiently raised liver transaminase levels. Following the above rationale, we have investigated the use of PVP in combination with Ce6 for application in lung cancers. As it was important to establish if cellular localization of Ce6-PVP was also exhibited in human lung tumor, the CAM tumor xenograft was employed here. We have demonstrated that this method of examining fluorescence uptake and retention in tissue explants on the CAM model provides a reliable means for direct, comparative visualization *in situ *of human tumors [20]. Inoculation of human NSCLC (NCI-H460) and SCLC (NCI-H526) into highly vascularized CAM led to the disseminated tumor growth on the surface of the CAM (Fig 2A, C). Typical fluorescence intensity image of NSCLC and SCLC are illustrated in Fig. 2B and 2D, respectively. Intense red fluorescence was macroscopically visible in the tumor cells under blue light, as compared to non-malignant epithelium of the CAM after 30 minutes post incubation with Ce6-PVP. The fluorescence retention by the lung tumor xenografts after topical administration was quantitatively evaluated using image-processing techniques and charted as a function of time (Fig. 3). High differential fluorescence intensity was observed between NSCLC xenografts and its surrounding normal CAM tissue compared to SCLC xenografts. The average of the red-to-blue intensity ratio of NSCLC xenograft was higher than that of SCLC xenograft. The fluorescence intensity elimination rate constant for NSCLC, SCLC and normal CAM was calculated to be 0.13, 0.25, and 0.38 min^-1 ^respectively, suggesting that Ce6-PVP is being retained longer in NSCLC than SCLC. Normal CAM had a faster elimination rate of Ce6-PVP.

**Figure 4:**
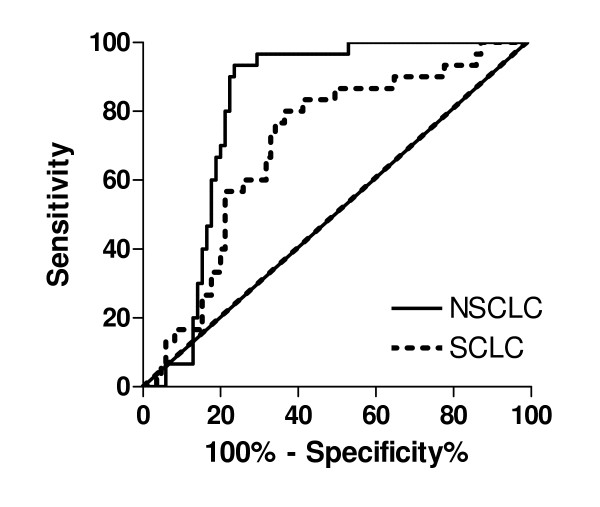
**Receiver operating characteristic curves illustrating the ability of Ce6-PVP to separate NSCLC (solid line) and SCLC (dotted line) from normal chorioallantoic membrane in the CAM model**. The ROC curve of two indistinguishable populations (i.e. abnormal versus normal region), represented by the 45-degree line (area under the ROC curve = 0.5), is included for comparison. Area under the ROC curve was 082 ± 0.04 (p < 0.0001) and 0.70 ± 0.05 (p = 0.0009118) for NSCLC and SCLC respectively.

We have applied ROC curve analysis from 0.5 to 5 h post administration of Ce6-PVP to validate the ability of the photosensitizer to discriminate NSCLC and SCLC from normal CAM membrane. The area under the curve (AUC) were then compared in order to make a fair judgment of the effectiveness of Ce6-PVP without being constricted to single values of sensitivity and specificity, which largely depend on the cut-off fluorescence intensity value chosen to distinguish normal from malignant region (Table 1). The following is a rough guide for classifying the accuracy of Ce6-PVP based on the AUC: 1 – 0.9 = excellent; 0.9 – 0.8 = good; 0.8 – 0.7 = fair; 0.7 – 0.6 = poor; and 0.6 – 0.5 = fail. The AUC for NSCLC were 0.52, 0.68, and 0.66, at 0.5 h, 1 h and 2 h respectively (P values were not statistically significant) indicating that shorter exposure times resulted in lower accuracy. The greatest AUC was observed from 3 h post drug administration onwards: *i.e. *0.88, 0.94 and 0.90 at 3 h, 4 h and 5 h respectively (all P values were statistically significant). For SCLC, the AUC were 0.52, 0.70, 0.68, 0.70, 0.74, and 0.58 at 0.5 h, 1 h, 2 h, 3 h, 4 h and 5 h respectively (P values were not statistically significant). This result showed no improvement in fluorescence accuracy in demarcating SCLC from the normal surrounding CAM. To evaluate the overall quality of fluorescence intensity discrimination between NSCLC and SCLC, a combined ROC was generated from 0.5 to 5 h post drug administration. The sensitivity and the specificity were calculated using different threshold (cut-off) values to distinguish healthy from malignant tissue (Fig 4). For NSCLC, the highest combined sensitivity and specificity were 90% and 78% (cut-off value > 4.0; likelihood ratio = 4.03), whereas for SCLC it was 57% and 79% respectively (cut-off value > 4.1; likelihood ratio = 2.68), implying that fluorescence mediated Ce6-PVP had distinctly higher rate of sensitivity for the detection of disseminated lesions of NSCLC than with SCLC.

**Table 1:** A comparison of areas under the ROC curves between NSCLC and SCLC at various time post drug administration.

Time post Ce6 – PVP administration, h	NSCLC		SCLC	
	
	Area under the ROC curve	P value	Area under the ROC curve	P value
0.5	0.52	0.9024	0.52	0.9025
1	0.68	0.2704	0.70	0.2207
2	0.66	0.3272	0.68	0.2704
3	0.88	0.0200*	0.70	0.2207
4	0.94	0.0071*	0.74	0.1417
5	0.90	0.0143*	0.58	0.6242

An area of 1 represents a perfect discrimination of tumor from normal tissue; an area of 0.5 represents no discrimination between normal and abnormal. The P value indicates whether the area under the ROC is significantly different from 0.5. *If the P value is < 0.05, the area under the ROC curve is significantly different (see description of statistical analysis). For tumors, each point represents a mean of 5 eggs whereas for normal, each point represents a mean of 10 eggs.

**Figure 5:**
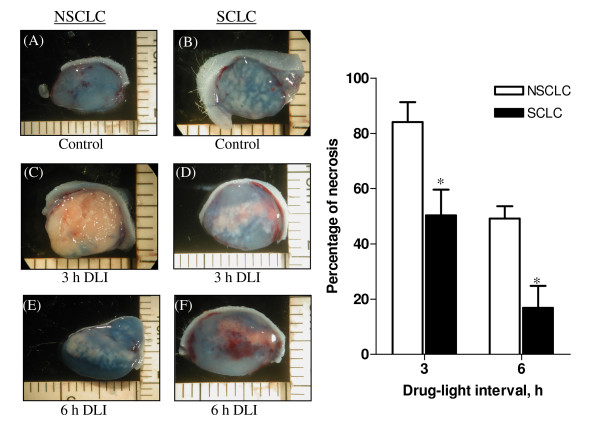
**A morphologic study of NSCLC and SCLC tumor damage efficiency using the method of vital staining with Evans blue at 48 h post Ce6-PVP mediated PDT**. Strong homogeneous staining was observed in the untreated controls (Fig. 5A, B), whereas in the treated tumor at 3 h drug-light interval (DLI) (Fig 5C, D) and at 6 h DLI (Fig 5E, F). Tissues damage was clearly distinguishable as an unstained area in the the tumor. Drug dose: 2.0 mg/kg; light dose: 150 J/cm^2^; 125 mW/cm^2^. Each data point is an average of at least 5 animals, Bars = standard error of the mean. *The mean difference is significant at the 0.05 level compared to the NSCLC group.

To determine the efficacy of Ce6-PVP mediated PDT, nude mice bearing NSCLC and SCLC tumors were administered with 2.0 mg/kg of the photosensitizer. PDT was performed on using light generated by a diode laser system (λ = 665 nm) at the light dose of 150 J/cm^2 ^and fluence rate of 125 mW/cm^2^. The area of tumor necrosis was measured by Evan's blue dye staining at 48 h post PDT. Strong heterogeneous staining was observed in the untreated controls (Fig. 5A, B) indicating occurrence of spontaneous, albeit limited necrosis, whereas in the PDT treated tumor, tissue damage was clearly evident as an unstained area (Fig 5C – F). NSCLC tumors irradiated at 3 and 6 h drug-light interval exhibited extent of tumor necrosis of 84 ± 7% and 50 ± 4% respectively. When PDT treatment was performed on SCLC models using the same parameter, it was observed that irradiation at 3 h drug-light interval resulted in 50 ± 9% of tumor necrosis while irradiation at 6 h drug-light interval resulted in 26 ± 8% tumor necrosis. Thus, we conclude that SCLC were only moderately sensitive to Ce6-PVP mediated PDT.

Almost all PDT studies were concerned with NSCLC due to the referral patterns in the clinics [5]. Although PDT has also been shown to be effective in the clinical treatment of SCLC [21], little preclinical data exist comparing the efficacy or tendency for resistance toward photosensitization between these two tumor histologies. In this study, we observed a certain degree of resistance to PDT in SCLC xenografts that could be related to faster elimination rate of Ce6-PVP, which resulted in lower cellular accumulation of the photosensitizer. There are already a variety of molecular markers that have been implicated in the pathogenesis of SCLC [22] thus making it difficult to hypothesize the molecular basis of acquired resistance towards photosensitization in our experiments. Generally, in lung cancer four types of multidrug resistance have been identified, i.e., classical multidrug resistance (MDR), non-P-glycoprotein MDR (also called MRP), atypical MDR (mediated through altered expression of topoisomerases II) and lung resistance-related protein [23]. Previous evidence indicates that SCLC cell lines and tumors express multidrug resistance-associated protein, i.e. MRP1, ATP binding cassette [ABC]C1 [24] and ABCG2 (ATP-binding cassette protein for breast cancer resistance protein) [25]. Hence, one plausible reason to explain the lack of activity of Ce6-PVP in SCLC is the possible existence of ABC transporters of chlorin-based (tetrapyrrole) photosensitizers in this tumor histology. The importance of human ABCG2 in the transport of tetrapyrrole structure has been implicated [26]. It was reported that cancer cell lines that expresses ABCG2 was found to efflux some of the chlorophyll based photosensitizers and thus may confer resistance to this treatment modality [27,28]. It has been suggested that by inhibiting ABCG2 transport using tyrosine kinase inhibitors (e.g. Gleevec), it is likely to be a more successful approach to enhancing clinical PDT [29].

## Conclusion

Photosensitization with Ce6-PVP for 3 hours of exposure time appeared to be most effective in detecting NSCLC in CAM model. Furthermore, PDT at 3 h drug-light interval resulted in a better tumor necrosis in NSCLC xenograft model. SCLC xenografts were found to manifest a certain degree of resistance to photosensitization with Ce6-PVP. Despite the limited activity of Ce6-PVP in the SCLC xenografts, it is conceivable that the combined modality of fluorescence imaging and targeted photodynamic therapy using Ce6-PVP may still have a potential role in SCLC. This warrants for additional studies on the molecular mechanisms of photosensitization resistance in SCLC to overcome this important clinical problem.

## Methods

### Photosensitizer: Ce6-PVP

Ce6-PVP was manufactured by ORPEGEN Pharma GmbH, Heidelberg, Germany. It is a co-lyophilisate of chlorin e6 (Ce6) and polyvinypyrrolidone (PVP, molecular mass ≈ 12,000) in a 1:1 mass ratio. A working concentration of 0.03 mM of Ce6-PVP was prepared using phosphate buffered saline to measure the absorption spectra. 1 ml of the solution was placed in a cuvette and the absorbance was measured from 300 – 800 nm. Absorption spectra were recorded on a spectrofluorophotometer RF-5301 PC (Shimadzu, Japan).

### Cell culture

The NCI-H460 cell line originates from human carcinoma of the large cell lung cancer. NCI-H526 cell lines originate from human carcinoma of the lung from the variant small cell lung cancer were obtained from the American Type Culture Collection, USA. NCI-H460 cells were cultured as a monolayer whereas NCI-H526 cells were cultured in suspension in RPMI-1640 medium supplemented with 10% fetal bovine serum, 1% non-essential amino acids (Gibco, USA), 1% sodium pyruvate (Gibco, USA), 100 units/ml penicillin/streptomycin (Gibco, USA) and incubated at 37°C, 95% humidity and 5% CO_2_. Before inoculation, the monolayer cells were washed with phosphate-buffered saline, trypsinized, and counted using a haemacytometer. Suspension cells were directly counted using a haemacytometer without trypsinization.

### CAM tumor xenograft

Fertilized chicken eggs were incubated at 37°C in a humidified atmosphere inside a hatching incubator equipped with an automatic rotator (Octagon 20, Brinsea, Somerset, UK). At embryo age (EA) 7, a window of about 1.5 cm was opened in the eggshell to detach the shell membrane from the developing CAM. Then, the window was sealed with sterilized parafilm to avoid contamination and the eggs were returned to the static incubator for further incubation until the day of experiments. On EA 9, approximately 5 × 10^6 ^NCI-H460 and NCI-H526 cells were inoculated on the CAM. The window of the eggs were resealed with sterile parafilm and returned to the static incubator. Grafted cells were allowed to grow on the CAM for up to 5 days. On EA 14, Ce6-PVP was dissolved in 0.9% sodium chloride (B. Braun Medical Inc, USA) to constitute a stock solution of 1 mg/mL. The stock solution was further diluted to obtain a volume of 80 μL containing a dose of 1 mg/kg body weight of the chick's embryo. The photosensitizer was applied on the entire surface of the CAM and left to incubate for 30 min. The window was resealed to avoid evaporation of the drug solution from the CAM. After 30 min incubation, imaging was performed at 0.5, 1, 2, 3, 4, 5, 6, and 24 h post drug administration. All procedures involving preparation and administration of the photosensitizer were conducted under low ambient lighting.

### Fluorescence imaging

Fluorescence images were performed using the Karl Storz D-light fluorescence endoscopy system (Karl Storz, Tuttlingen, Germany). This D-light system consisted of xenon short arc lamp, filtered by a band pass filter (370 – 450 nm) to excite Ce6-PVP and a sensitive colour CCD video camera connected to a modified endoscope integrated with long pass filter (cut-off wavelength at 560 nm). The red channel registered the photosensitizer's fluorescence and the blue channel captured the diffusely back-scattered excitation light. The intensities of the red and blue channels of the fluorescence images were quantified using the software MicroImage (Olympus Optical Co. (Europa), Germany). The red-to-blue intensity ratio of the fluorescence endoscopic image algorithm was found to be effective in separating benign tissue from dysplasia, and carcinoma in situ/squamous cell carcinoma from dysplasia [30]. By applying the red-to-blue intensity ratio as a diagnostic algorithm, the intensities of the red fluorescence of Ce6-PVP are determined as a function of time. Such algorithm is independent of the geometries of excitation/collection of signals and the power of excitation during the fluorescence imaging process [31].

### Statistical analysis of fluorescence image

To statistically evaluate the temporal fluorescence of Ce6-PVP, logistic regression and receiver operating characteristics (ROC) curve [32] was determined using the GraphPad software for Windows (GraphPad, San Diego, CA). The elimination rate constant of Ce6-PVP was calculated by a method fitting the data to a one-phase exponential decay equation. The validity of fitted curve was verified with the test of runs (F test) in each case. Area under the curve (AUC), P value, and cut-off point were obtained from the ROC curve. The area under the ROC curve measures accuracy of the fluorescence images. The accuracy of the ROC curve analysis is based on how well the fluorescence images discriminates the tumor region from the normal CAM, as defined by white light imaging. The closer the curve follows the left border and then the top border of the ROC space, the more accurate the test. The closer the curve comes to the 45-degree diagonal of the ROC space, the less accurate the test. In addition, likelihood ratios (where the likelihood ratio is defined as the ratio of the probability of the fluorescence signal for tumor to the probability of the fluorescence signal for normal region) were calculated to help determine the 'best' cut-off point to compare sensitivity and specificity between NSCLC and SCLC.

### PDT treatment on murine xenograft model

Male Balb/c athymic (nu+/nu+) (ARC, WA, Australia) mice were used for tumor xenografting at the age of 8–10 weeks. Approximately 3.0 × 10^6 ^NCI-H460 and NCI-H526 cells suspended in 150 μl of Hank's buffered saline solution were injected subcutaneously into both lower flanks of the mice. The animals were used for experiments when the tumors measured a surface diameter around 7 – 10 mm. A dose of 2.0 mg/kg of the photosensitizer was administered intravenously through the tail vein. The mice were anaesthetized with 50 μl cocktail of ketamine hydrochloride (50 mg/ml, Trittau, Germany) and valium (1:1 vol/vol) through intraperitoneal injection. A diode laser (Ceralas PDT 665, Biolitec) emitting at a wavelength of 665 ± 3 nm was used for irradiation. The peak power output was calibrated to 1.65 W at the fiber tip before commencement of irradiation. The laser energy with a total fluence of 150 J/cm^2 ^was delivered to a 1.0 cm^2 ^circular spot on the surface of the tumor via a silica fiber frontal light distributor (FD model, Medlight, Switzerland). Fluence rate of 125 mW/cm^2 ^was measured using a power meter (LaserCheck, Coherent, USA). PDT treatment was performed at 3 and 6 h drug-light interval on one tumor while the contralateral tumor that was not irradiated served as controls. All procedures were approved by the national experimental animal welfare institution (Institutional Animal Care and Use Committee, SingHealth, Singapore), in accordance with international standards.

### Macroscopic assessment of tumor response post PDT

At 48 h post PDT, 1% Evans Blue (Merck, Germany) in PBS was injected intraperitoneally at a volume of 0.4 mL in mice for examination of viable or necrotic tissues at post PDT. Six hours later, animals were sacrificed and the tumors were excised. Around 2–3 mm thick cross-section slices were cut in a plane parallel to the direction of incident light and imaged under a stereoscopic microscope (Stemi 2000C, Zeiss, Germany). The unstained area was attributed to tissue necrosis, whereas the blue stained area indicated viable tissue. Digital images were all analyzed using NIH Image v1.62 software. Each image captured had the same calibration values to allow uniformity in the processing of the images. The tumor was outlined using the freehand drawing tool to measure the total tumor area. Similarly the necrotic area of the tumor was measured. The percentage of necrosis was calculated as the necrotic area divided by the total tumor area multiplied by 100. Statistical analysis (Student's t test) was used for multiple comparisons. The criterion for statistical significance was set at the 0.05 level.

## Authors' contributions

WWC, PWH and MO conceived of the study, and participated in its design and coordination. All authors read and approved the final manuscript
